# Anomalous Case of Malformation in a Child-bearing Woman

**Published:** 1848-04

**Authors:** John Smith

**Affiliations:** Kingston, Luzerne county, Pennsylvania


*Anomalous Case of Malformation in a child-bearing woman.* By John Smith, M. D., of Kingston, Luzerne county, Pennsylvania.

June 27th, 1844, I was called to attend a woman above the medium size, in her first labour; I found her pains regular, the second stage fully established. On attempting to pass my finger to ascertain the presenting part, I encountered, external to the labia, a membrane containing a fluid, which I supposed at the time to be the bag of waters of the ovum; as it did not expand at the next expulsive effort, and presenting a feeling different from the ordinary membrane, I passed my finger by it into the vagina, found the vertex presenting in the most favourable position, with the os uteri dilated about two inches in diameter, and the waters of the ovum already discharged. In the absence of pain, I passed my finger round the os uteri, and found that the protruding part had no connection with it, but was connected with or passed through the vagina, a little to the left of the middle and near the superior part of the pubis. As my patient had several times complained of what I understood to be simply a bearing down, usual with many females in a state of gestation, I was a little surprised to find a malformation of the kind. To my enquiries she answered that previous to her marriage, there was never any thing made its appearance external to the labia, but since she became enciente, and more particularly for the last few months, whenever in an erect posture, either sitting or standing, (to use her own expression) it was continually in the world, and that she had frequently an uneasy sensation in the region of the left ovarium. I informed my patient that there was something not natural to the female, but that it would not interfere with her labour or impede the passage of the child. She however expressed some uneasiness, and I had my friend Dr. T. W. Minor called in, who, after making a careful examination, confirmed the statements I had made, and retired. In a little more than an hour from my first being called, my patient was comfortably in bed. After the passing of the placenta, I returned the protruding sack within the vagina, and her recovery was as favourable as could be expected from any ordinary labour ; but immediately on her rising from bed, the sack with its contents protruded again, and was a source of much inconvenience. I now examined it by inspection, and found it to consist of a membrne having numerous small blood vessels on and near its surface, running in various directions, presenting all the external appearance of the scrotum of the male, and on making slight traction it always occasioned pain and uneasiness in the vicinity of the left ovarium. After introducing a gum catheter, to establish the fact clearly that it was wholly unconnected with the urinary bladder, I punctured it with a crown lancet, and drew off about two ounces of limpid serum. No benefit was derived from this simple operation. It did not retract within the vagina, while the irritation produced in walking prevented the puncture from healing; it became corrugated, thickened, and pus formed. I now, with a pair of scissors, severed the protruding portion of the sack; the remainder retired to the superior part of the pubis, where I could distinctly feel the cut edges. After the operation no treatment was required, nor was my patient confined to her bed at all. A slight soreness above the pubes on the left side was felt for a few days.

Feb. 11th, 1846, I attended the same female in her second labour. On making my first examination, I could find no mark of there ever having been any malformation; her labour was favourable and she is now in good health.

Was this malformation a sack connected with the left ovarium?

